# Supplementary material for: Urban–Rural Disparities in Case Fatality of Community-Acquired Sepsis in Germany: A Retrospective Cohort Study
Source: Int J Environ Res Public Health. 2023 May 18;20(10):5867. doi: 10.3390/ijerph20105867 (PMC10218320; doi:10.3390/ijerph20105867)
Supplement: Supplementary file 1 [file ijerph-20-05867-s001.zip › ijerph-2366514-supplementary.docx]

*Article*

**Urban-rural disparities in case fatality of community-acquired sepsis in Germany: a retrospective cohort study.**

**Claudia Matthaeus-Kraemer ^1,2,*^, Norman Rose ^1,2,*^, Melissa Spoden ^3^, Mathias Pletz ^1^, Konrad Reinhart ^4^ and Carolin Fleischmann-Struzek ^1,2^**

^1^ Institute of Infectious Diseases and Infection Control, Jena University Hospital, Jena, Germany

^2^ Center for Sepsis Control and Care, Jena University Hospital, Jena, Germany

^3^ Wissenschaftliches Institut der Ortskrankenkassen, Berlin, Germany

^4^ Charité-Universitätsmedizin Berlin, Department of Anesthesiology and Operative Intensive Care, Berlin, Germany

* These authors contributed equally to this work.

Correspondence: carolin.fleischmann-struzek@med.uni-jena.de

Content Page

Definitions of urban and rural regions 2

Case identification: Definitions and Codes 3

Characteristics of sepsis and index treatment 4

**Definitions of urban and rural regions^1^**

In Germany, different district types of settlement structure defined by the “Federal Institute for Research on Building, Urban Affairs and Spatial Development”. They are characterized by:

- Proportion of population in large and medium-sized cities
- Presence and size of a large city
- Population density in regions in general
- Population density without consideration of large cities

Following these criteria, regions were categorized in:

1. Urban regions: Regions where at least 50% of the population lives in large and medium-sized cities and where is a large city with around 500,000 inhabitants and more as well as regions with a population density of at least 300 inhabitants / km² without consideration of large cities.
2. Regions with urban character / semiurban regions: Regions where at least 33% of the population lives in large and medium-sized cities with a population density between 150 and 300 Inhabitants/km² as well as regions with at least one large city and which have a population density of at least 100 inhabitants/km² without consideration of large cities.
3. Rural regions: Regions where less than 33% of the population lives in large and medium-sized cities with a population density below 150 inhabitants / km².

**Case identification: Definitions and Codes**

The identification of sepsis patients in our study was based on the following ICD-10-GM codes in health claims data. At least one of the codes had to be coded as primary and secondary discharge diagnosis. During the complete observation period, sepsis coding was based on the sepsis-1/2 criteria in Germany. Therefore, severity was classified as: sepsis – all forms; severe sepsis (including septic shock); septic shock; and non-severe sepsis.

| Sepsis |  |
| --- | --- |
| ICD-10-GM Codes: |  |
| A02.1 | Salmonella sepsis |
| A20.0 | Bubonic plague |
| A20.7 | Septicaemic plague |
| A21.7 | Generalized tularaemia |
| A22.7 | Anthrax sepsis |
| A24.1 | Acute or fulminating melioidosis |
| A26.7 | Erysipelothrix sepsis |
| A28.2 | Extraintestinal yersiniosis |
| A32.7 | Listerial sepsis |
| A39.1 | Waterhouse-Friderichsen syndrome |
| A39.2 | Acute meningococcal sepsis |
| A39.3 | Chronic meningogoccal sepsis |
| A39.4 | Meningococcaemia, unspecified |
| A40 | Streptococcal sepsis |
| A41 | Other sepsis |
| A42.7 | Actinomycotic sepsis |
| A48.3 | Toxic shock syndrome |
| A49.9 | Bacterial infection, unspecified |
| A54.8 | Other gonococcal infections |
| B00.7 | Disseminated herpesviral disease |
| B37.6 | Candidal endocarditis |
| B37.7 | Candidal sepsis |
| B49 | Unspecified mycosis |
| O75.3 | Other infection during labour |
| O85 | other puerperal infections |
| R65.0 | Systemic Inflammatory Response Syndrome of infectious origin without organ failure |
| R65.1 | Systemic Inflammatory Response Syndrome of infectious origin with organ failure |
| R57.2 | Septic shock |

| Severe sepsis |  |
| --- | --- |
| ICD-10-GM Codes: |  |
| R65.1 | Systemic Inflammatory Response Syndrome of infectious origin with organ failure |

| Septic shock |  |
| --- | --- |
| ICD-10-GM Codes: |  |
| R57.2 | Septic shock |

**Characteristics of sepsis and index treatment**

Assessed at discharge from the index treatment

| **Focus of infection** |  |
| --- | --- |
| Respiratory tract |  |
| ICD-10-GM Codes: |  |
| J01 | Acute sinusitis |
| J02 | Acute pharyngitis |
| J03 | Acute tonsillitis |
| J04 | Acute laryngitis and tracheitis |
| J06 | Acute upper respiratory infections of multiple and unspecified sites |
| J05 | Acute obstructive laryngitis [croup] and epiglottitis |
| J09 | Influenza due to identified zoonotic or pandemic influenza virus |
| J10 | Influenza due to identified seasonal influenza virus |
| J11 | Influenza, virus not identified |
| J12 | Viral pneumonia, not elsewhere classified |
| J13 | Pneumonia due to Streptococcus pneumoniae |
| J14 | Pneumonia due to Haemophilus influenzae |
| J15 | Bacterial pneumonia, not elsewhere classified |
| J16 | Pneumonia due to other infectious organisms, not elsewhere classified |
| J17 | Pneumonia in diseases classified elsewhere |
| J18 | Pneumonia, organism unspecified |
| J20 | Acute bronchitis |
| J21 | Acute bronchiolitis |
| J22 | Unspecified acute lower respiratory infection |
| J44.0 | Chronic obstructive pulmonary disease with acute lower respiratory infection |
| J44.1 | Chronic obstructive pulmonary disease with acute exacerbation, unspecified |
| J86 | Pyothorax |
| J85 | Abscess of lung and mediastinum |
| A15 | Respiratory tuberculosis, bacteriologically or histologically confirmed |
| A16 | Respiratory tuberculosis, not confirmed bacteriologically or histologically |
| U69.00 | Hospital-acquired pneumonia in other diseases classified elsewhere |
| A36 | Diphtheria |
| A37 | Whooping cough |
| B38 | Coccidioidomycosis |
| B39 | Histoplasmosis |

| Abdominal infections |  |
| --- | --- |
| ICD-10-GM Codes: |  |
| A00 | Cholera |
| A01 | Typhoid and paratyphoid fevers |
| A02 | Other salmonella infections |
| A03 | Shigellosis |
| A04 | Other bacterial intestinal infections |
| A05 | Other bacterial foodborne intoxications, not elsewhere classified |
| A06 | Amoebiasis |
| A07 | Other protozoal intestinal diseases |
| A08 | Viral and other specified intestinal infections |
| A09 | Other gastroenteritis and colitis of infectious and unspecified origin |
| K35 | Acute appendicitis |
| K37 | Unspecified appendicitis |
| K36 | Other appendicitis |
| K57.02 | Diverticular disease of small intestine with perforation and abscess without bleeding |
| K57.03 | Diverticular disease of small intestine with perforation and abscess with bleeding |
| K57.12 | Diverticular disease of small intestine without perforation or abscess without bleeding |
| K57.13 | Diverticular disease of small intestine without perforation or abscess wit bleeding |
| K57.22 | Diverticular disease of large intestine with perforation and abscess without bleeding |
| K57.23 | Diverticular disease of large intestine with perforation, abscess and bleeding |
| K57.32 | Diverticular disease of large intestine without perforation or abscess wihout bleeding |
| K57.33 | Diverticular disease of large intestine without perforation or abscess wit bleeding |
| K57.42 | Diverticular disease of both small and large intestine with perforation and abscess without bleeding |
| K57.43 | Diverticular disease of both small and large intestine with perforation, abscess and bleeding |
| K57.52 | Diverticular disease of both small and large intestine without perforation or abscess or bleeding |
| K57.53 | Diverticular disease of both small and large intestine without perforation or abscess with bleeding |
| K57.82 | Diverticular disease of intestine, part unspecified, with perforation and abscess without bleeding |
| K57.83 | Diverticular disease of intestine, part unspecified with perforation, abscess and bleeding |
| K57.92 | Diverticular disease of intestine, part unspecified, without perforation, abscess or bleeding |
| K57.93 | Diverticular disease of intestine, part unspecified, without perforation or abscess with bleeding |
| K61 | Abscess of anal and rectal regions |
| K65 | Peritonitis |
| K67 | Disorders of peritoneum in infectious diseases classified elsewhere |
| K63.0 | Abscess of intestine |
| K63.1 | Perforation of intestine (nontraumatic) |
| K75.0 | Abscess of liver |
| K75.1 | Phlebitis of portal vein |
| K81.0 | Cholecystitis |
| K77.0 | Liver disorders in infectious and parasitic diseases classified elsewhere |
| U69.40! | Recurrent infection due to Clostridium difficile |

| Wound/soft tissue infection |  |
| --- | --- |
| ICD-10-GM Codes: |  |
| A46 | Erysipelas |
| B47 | Mycetoma |
| L03 | Phlegmon |
| L04 | Acute lymphadenitis |
| L08 | Other local infections of skin and subcutaneous tissue |
| L05 | Pilonidal cyst |
| B00 | Herpesviral [herpes simplex] infections |
| B07 | Viral warts |
| B08 | Other viral infections characterized by skin and mucous membrane lesions, not elsewhere classified |
| B09 | Unspecified viral infection characterized by skin and mucous membrane lesions |
| H05.0 | Acute inflammation of orbit |
| H60.2 | Malignant otitis externa |
| H70.0 | Acute mastoiditis |
| J36 | Peritonsillar abscess |
| J39.0 | Retropharyngeal and parapharyngeal abscess |
| J39.1 | Other abscess of pharynx |
| L02 | Cutaneous abscess, furuncle and carbuncle |

| Genitourinary system infection |  |
| --- | --- |
| ICD-10-GM Codes: |  |
| N10 | Acute tubulo-interstitial nephritis |
| N15.1 | Renal and perinephric abscess |
| N15.9 | Renal tubulo-interstitial disease, unspecified |
| N34 | Urethritis and urethral syndrome |
| N30 | Cystitis |
| N39.0 | Urinary tract infection, site not specified |
| N41 | Inflammatory diseases of prostate |
| N45 | Orchitis and epididymitis |
| N48.2 | Other inflammatory disorders of penis |
| N49 | Inflammatory disorders of male genital organs, not elsewhere classified |
| N70 | Salpingitis and oophoritis |
| N71 | Inflammatory disease of uterus, except cervix |
| N72 | Inflammatory disease of cervix uteri |
| N73 | Other female pelvic inflammatory diseases |
| N74 | Female pelvic inflammatory disorders in diseases classified elsewhere |
| N75 | Diseases of Bartholin gland |
| N76 | Other inflammation of vagina and vulva |
| N77 | Vulvovaginal ulceration and inflammation in diseases classified elsewhere |
| N61 | Inflammatory disorders of breast |
| N98.0 | Infection associated with artificial insemination |
| A59 | Trichomoniasis |
| A55 | Chlamydial lymphogranuloma (venereum) |
| A56 | Other sexually transmitted chlamydial diseases |

| Central nervous system infection |  |
| --- | --- |
| ICD-10-GM Codes: |  |
| A39 | Meningococcal infection |
| G00 | Bacterial meningitis, not elsewhere classified |
| G01 | Meningitis in bacterial diseases classified elsewhere |
| G02 | Meningitis in other infectious and parasitic diseases classified elsewhere |
| G03 | Meningitis due to other and unspecified causes |
| G04 | Encephalitis, myelitis and encephalomyelitis |
| G05* | Encephalitis, myelitis and encephalomyelitis in diseases classified elsewhere |
| G06 | Intracranial and intraspinal abscess and granuloma |
| G07* | Intracranial and intraspinal abscess and granuloma in diseases classified elsewhere |
| G08 | Intracranial and intraspinal phlebitis and thrombophlebitis |
| A17+ | Tuberculosis of nervous system |
| A81 | Atypical virus infections of central nervous system |
| A83 | Mosquito-borne viral encephalitis |
| A84 | Tick-borne viral encephalitis |
| A85 | Other viral encephalitis, not elsewhere classified |
| A86 | Unspecified viral encephalitis |
| A87 | Viral meningitis |
| A88 | Other viral infections of central nervous system, not elsewhere classified |
| A89 | Unspecified viral infection of central nervous system |

| Cardiovascular system infection |  |
| --- | --- |
| ICD-10-GM Codes: |  |
| I32 | Pericarditis in diseases classified elsewhere |
| I33 | Acute and subacute endocarditis |
| I39 | Endocarditis and heart valve disorders in diseases classified elsewhere |
| I40 | Acute myocarditis |
| I41 | Myocarditis in diseases classified elsewhere |
| I80 | Thombosis, phlebitis and thrombophlebitis |
| I38 | Endocarditis, valve unspecified |
| I98.1 | Cardiovascular disorders in other infectious and parasitic diseases classified elsewhere |

| Device-related infections |  |
| --- | --- |
| ICD-10-GM Codes: |  |
| T82.6 | Infection and inflammatory reaction due to cardiac valve prosthesis |
| T82.7 | Infection and inflammatory reaction due to other cardiac and vascular devices, implants and grafts |
| T83.5 | Infection and inflammatory reaction due to prosthetic device, implant and graft in urinary system |
| T83.6 | Infection and inflammatory reaction due to prosthetic device, implant and graft in genital tract |
| T84.5 | Infection and inflammatory reaction due to internal joint prosthesis |
| T84.6 | Infection and inflammatory reaction due to internal fixation device [any site] |
| T84.7 | Infection and inflammatory reaction due to other internal orthopaedic prosthetic devices, implants and grafts |
| T85.7 | Infection and inflammatory reaction due to other internal prosthetic devices, implants and grafts |

| Pregnancy associated infection |  |
| --- | --- |
| ICD-10-GM Codes: |  |
| O75.3 | Other infection during labour |
| O85 | Puerperal fever |
| O03.0 | Spontaneous abortion, complicated by genital tract and pelvic infection, incomplete |
| O03.5 | Spontaneous abortion, complicated by genital tract and pelvic infection, complete or unspecified |
| O04.0 | Medical abortion, complicated by genital tract and pelvic infection, incomplete |
| O04.5 | Medical abortion, complicated by genital tract and pelvic infection, complete or unspecified |
| O05.0 | Other abortion, complicated by genital tract and pelvic infectio, incomplete |
| O05.5 | Other abortion, complicated by genital tract and pelvic infection, complete or unspecified |
| O06.0 | Unspecified abortion, complicated by genital tract and pelvic infection, incomplete |
| O06.5 | Unspecified abortion, complicated by genital tract and pelvic infection, complete or unspecified |
| O07.0 | Failed medical abortion, complicated by genital tract and pelvic infection |
| O07.5 | Other and unspecified failed attempted abortion, complicated by genital tract and pelvic infection |
| O08.0 | Genital tract and pelvic infection following abortion and ectopic and molar pregnancy |
| O86 | Other puerperal infections |
| O23 | Infections of genitourinary tract in pregnancy |
| O41.1 | Infection of amniotic sac and membranes |
| O88.3 | Obstetric pyaemic and septic embolism |
| O91 | Infections of breast associated with childbirth |
| O98 | Maternal infectious and parasitic diseases classifiable elsewhere but complicating pregnancy, childbirth and the puerperium |

| Hospital-acquired infections |  |
| --- | --- |
| ICD-10-GM Codes: |  |
| T82.6 | Infection and inflammatory reaction due to cardiac valve prosthesis |
| T82.7 | Infection and inflammatory reaction due to other cardiac and vascular devices, implants and grafts |
| T84.5 | Infection and inflammatory reaction due to internal joint prosthesis |
| T84.6 | Infection and inflammatory reaction due to internal fixation device [any site] |
| T84.7 | Infection and inflammatory reaction due to other internal orthopaedic prosthetic devices, implants and grafts |
| T85.72 | Infection and inflammatory reaction due to internal prosthetic devices, implants and grafts in the central nervous system |
| T85.73 | Infection and inflammatory reaction due to prosthetic devices or implants of the mamma |
| T85.75 | Infection and inflammatory reaction due to internal prosthetic devices, implants or grafts of the hepatobiliary system or pancreas |
| T85.76 | Infection and inflammatory reaction due to internal prosthetic devices, implants or grafts of the other gastrointestinal system |
| T85.78 | Infection and inflammatory reaction due to other internal prosthetic devices, implants and grafts |
| O86.0 | Infection of obstetric surgical wound |
| T83.5 | Infection and inflammatory reaction due to prosthetic device, implant and graft in urinary system |
| T83.6 | Infection and inflammatory reaction due to prosthetic device, implant and graft in genital tract |
| A04.7 | Enterocolitis due to Clostridium difficile |
| U69.40! | Recurrent infection due to Clostridium difficile |
| T80.2 | Infections following infusion transfusion and therapeutic injection |
| T82.7 | Infection and inflammatory reaction due to other cardiac and vascular devices, implants and grafts |
| T81.4 | Infection following a procedure, not elsewhere classified |
| T85.71 | Infection and inflammatory reaction due to peritoneal dialysis catheter |
| T85.74 | Infection and inflammatory reaction due to percutaneous endoscopic gastrostomy/jejunostomy,T88.0 |
| U69.00 | Hospital-acquired pneumonia in patients aged 18 years or older |

| Multidrug-resistant infections |  |
| --- | --- |
| ICD-10-GM Codes: |  |
| U80.! | Grampositive bacteria with specified antibiotic resistance, requiring special therapeutic or hygienic measures |
| U81.! | Gram negative bacteria with specified antibiotic resistanc, requiring special therapeutic or hygienic measures |
| U82.! | Mycobacteria with resistance against TB drugs (first line) |
| U83.! | Candida with resistance against Fluconazole and Voriconazole |
| U84.! | Herpes virus with restistance against antivirals |
| U85! | Human Immunodeficiency Virus with resistance against antivirals or proteinase â€“ inhibitors |
| OPS Codes: |  |
| 8-987 | Complex treatment in the case of colonisation or infection with multidrug-resistant pathogens [MDR] |

| **Organ dysfunctions** |  |
| --- | --- |
| Cardiovascular dysfunction/shock |  |
| ICD-10-GM Codes: |  |
| I95.9 | Hypotension, unspecified |
| R57.8 | Other shock |
| R57.9 | Shock, unspecified |
| R57.2 | Septic shock |

| Respiratory dysfunction |  |
| --- | --- |
| ICD-10-GM Codes: |  |
| J96 | Respiratory failure, not elsewhere classified |
| J80 | Adult respiratory distress syndrome |
| J98.4 | Other disorders of lung |
| R06.0 | Dyspnoea |
| R06.8 | Other and unspecified abnormalities of breathing |

| Encephalopathy |  |
| --- | --- |
| ICD-10-GM Codes: |  |
| F05 | Delirium, not induced by alcohol and other psychoactive substances |
| G93.1 | Anoxic brain damage, not elsewhere classified |
| G93.4 | Encephalopathy, unspecified |
| R40 | Somnolence, stupor and coma |

| Renal dysfunction |  |
| --- | --- |
| ICD-10-GM Codes: |  |
| N17. | Acute renal failure |
| N19 | Unspecified kidney failure |

| Metabolic dysfunction |  |
| --- | --- |
| ICD-10-GM Codes: |  |
| E87.2 | Acidosis |

| Abnormal coagulation |  |
| --- | --- |
| ICD-10-GM Codes: |  |
| Coagulation D65 | Disseminated intravascular coagulation [defibrination syndrome] |
| D68.8 | Other specified coagulation defects |
| D68.9 | Coagulation defect, unspecified |
| D69.5 | Secondary thrombocytopenia |
| D69.6 | Thrombocytopenia, unspecified |

| Hepatic dysfunction |  |
| --- | --- |
| ICD-10-GM Codes: |  |
| K72.0 | Acute and subacute hepatic failure |
| K72.7 | Hepatic encephalopathy and hepatic coma |
| K72.9 | Hepatic failure, unspecified |
| K76.2 | Central haemorrhagic necrosis of liver |
| K76.3 | Infarction of liver |

| Other organ dysfunction |  |
| --- | --- |
| ICD-10-GM Codes: |  |
| R65.1 | Systemic Inflammatory Response Syndrome of infectious origin with organ complications |

| ICU Treatment |  |
| --- | --- |
| OPS Codes: |  |
| 8-980 | Intensive care complex treatment |
| 8-98f | Costly intensive care complex treatment (basic procedure) |
| 8-98d | Intensive care complex treatment in childhood (basic procedure) |
| 8-98c | Intensive care complex treatment in childhood |

| Mechanical Ventilation |  |
| --- | --- |
| OPS Codes: |  |
| 8-713 | Mechanical ventilation and respiratory support in adults |
| 8-712 | Mechanical ventilation and respiratory support in children and adolescents |
| 8-714 | Special procedure for mechanical ventilation in the case of severe respiratory failure |
| 8-70 | Access for mechanical ventilation and measures to maintain the airway |
| 8-71 | Mechanical ventilation and respiratory support via a mask or tube and ventilation weaning |

| Renal replacement therapy |  |
| --- | --- |
| OPS Codes: |  |
| 8-853 | Haemofiltration |
| 8-854 | Haemodialysis |
| 8-855 | Haemodiafiltration |
| 8-857 | Peritoneal dialysis |
| 8-85a | Dialysis procedure due to a functional failure and failure of a kidney transplant |

| Tracheostomy during hospitalization |  |
| --- | --- |
| OPS Codes: |  |
| 5-311 | Temporary tracheostomy |
| 5-312 | Permanent tracheostomy |
| Surgical treatment |  |
| OPS Codes: |  |
| Any OPS Code from Chapter 5 |  |

| Amputation during treatment |  |
| --- | --- |
| OPS Codes: |  |
| 5-862 | Amputation and exarticulation of upper extremity |
| 5-863 | Amputation and exarticulation of hand |
| 5-864 | Amputation and exarticulation of lower extremity |
| 5-865 | Amputation and exarticulation of foot |
| 5-866 | Revision of amputation area |

| Palliative care |  |
| --- | --- |
| OPS Codes: |  |
| 8-982 | Palliative medical complex treatment |
| 8-98e | Specialized inpatient palliative medical complex treatment |
| 8-98h | Specialized palliative medical complex treatment through a palliative care service |

| Early rehabilitation treatment |  |
| --- | --- |
| OPS Codes: |  |
| 8-55 | Interdisciplinary and other early rehabilitation |

| **Discharge disposition** | **Definition** |
| --- | --- |
| regular | regular termination of treatment, with or without post-discharge treatment intended |
| other hospital | transfer to another hospital;  transfer to another hospital as part of a cooperation;  external transfer for psychiatric treatment |
| hospice | discharge into a hospice |
| rehabilitation | discharge into a rehabilitation facility |
| nursing home | discharge into a long-term care facility |
| other | treatment terminated for other reasons, with or without post-discharge treatment intended;  Treatment terminated against medical advice, with or without post-discharge treatment intended;  Change of responsibility of the cost bearer;  Death;  internal routing;  Treatment terminated for other reasons, post-inpatient treatment  intended;  external transfer with relocation or change between the  Remuneration ranges of the DRG flat rate case, according to section 17b (1) first sentence of the Hospital Funding Act;  Internal transfer with a change between the DRG fee ranges  according to section 17b (1)first sentence of the Hospital Funding Act;  Relocation;  Discharge before resumption with reclassification;  Discharge before resumption with reclassification due to complication;  Discharge or transfer with subsequent readmission;  Case closure (internal transfer) when changing between full,  day-care and ward-equivalent treatment;  Start of an outside stay with an absence past midnight  (BPflV area - for the specialist department for laying);  Ending an outside stay with an absence past midnight  (BPflV area - for pseudo specialist department 0003);  Discharge at the end of the year if accepted in the previous year (for the purposes of  Billing - § 4 PEPPV);  Beginning of a period without direct patient contact  (station equivalent treatment);  Termination of a period without direct patient contact  (ward equivalent treatment - for pseudo-specialist department 0004); |

**Comorbidities**

defined according to Charlson Comorbidity Index^2^

| Pre-existing immobility |  |
| --- | --- |
| ICD-10-GM Codes: |  |
| R26.2 | Difficulty in walking, not elsewhere classified |
| R26.3 | Immobility |
| R29.6 | Tendency to fall, not elsewhere classified |
| Z99.3 | Dependence on wheelchair |
| Z74.0 | Need for assistance due to reduced mobility |

| Pre-existing long-term mechanical ventilation |  |
| --- | --- |
| ICD-10-GM Codes: |  |
| Z99.0 | Dependence on aspirator |
| Z99.1 | Dependence on respirator |
| OPS Codes: |  |
| 8-713 | Mechanical ventilation and respiratory support in adults |
| 8-712 | Mechanical ventilation and respiratory support in children and adolescents |
| 8-714 | Special procedure for mechanical ventilation in the case of severe respiratory failure |
| 8-70 | Access for mechanical ventilation and measures to maintain the airway |
| 8-71 | Mechanical ventilation and respiratory support via a mask or tube and ventilation weaning |

| Pre-existing dialysis |  |
| --- | --- |
| ICD-10-GM Codes: |  |
| Z99.2 | Dependence on renal dialysis |
| Z49 | Care involving dialysis |
| OPS Codes: |  |
| 5-392 | Creation of an arteriovenous fistula |
| 8-853 | Haemofiltration |
| 8-854 | Haemodialysis |
| 8-855 | Haemodiafiltration |
| 8-857 | Peritoneal dialysis |
| Statutory scale of fees for physicians (GOÄ) Codes: |  |
| 13602 | Flat rate supplementary fee for continuous care of a patient requiring dialysis |
| 13610 | Flat rate supplementary fee for medical care in the case of haemodialysis, peritoneal dialysis and special procedures |
| 13611 | Flat rate supplementary fee for medical care in the case of peritoneal dialysis |
| 4562 | Flat rate supplementary fee for continuous care of a patient requiring dialysis |
| 4564 | Flat rate supplementary fee for paediatric nephrology care when carrying out haemodialysis |
| 4565 | Flat rate supplementary fee for paediatric nephrology care when carrying out peritoneal dialysis |
| 40815 | Flat rate fee for dialysis in patients up to the age of 18 years at their place of residence |
| 40816 | Flat rate fee for peritoneal dialysis in patients up to the age of 18 years |
| 40817 | Flat rate fee for peritoneal dialysis in patients up to the age of 18 years at their place of residence |
| 40818 | Flat rate fee for haemodialysis in patients up to the age of 18 years during a holiday or other absence |
| 40819 | Flat rate fee for peritoneal dialysis in patients up to the age of 18 years during a holiday or other absence |
| 40823 | Flat rate fee for dialysis in insured persons from the age of 18 years |
| 40824 | Flat rate fee for dialysis in insured persons from the age of 18 years at their place of residence |
| 40825 | Flat rate fee for peritoneal dialysis in insured persons from the age of 18 years |
| 40826 | Flat rate fee for peritoneal dialysis in insured persons from the age of 18 years at their place of residence |
| 40827 | Flat rate fee for intermittent peritoneal dialysis in insured persons from the age of 18 years at their place of residence |
| 40828 | Flat rate fee for dialysis from the age of 18 years during a holiday or work-related stay |
| 40829 | Supplement to flat rate fee 40823 or 40825 for insured persons aged 59-69 years |
| 40830 | Supplement to flat rate fee 40824, 40826 and 40827 for insured persons aged 59-69 years |
| 40831 | Supplement to flat rate fee 40823 or 40825 for insured persons aged 69-79 years |
| 40832 | Supplement to flat rate fee 40824, 40826 and 40827 for insured persons aged 69-79 years |
| 40833 | Supplement to flat rate fee 40823 or 40825 for insured persons from 79 years of age |
| 40834 | Supplement to flat rate fee 40824, 40826 and 40827 for insured persons from 79 years of age |
| 40835 | Supplement to flat rate fee 40816, 40823 or 40825 for dialysis in a patient with an infection |
| 40836 | Supplement to flat rate fee 40815, 40817, 40818, 40819, 40824, 40826 to 40828 for dialysis in a patient with an infection |
| 40837 | Supplement to flat rate fee 40816 or 40825 for intermittent peritoneal dialysis |
| 40838 | Supplement to flat rate fee 40817, 40819, 40827 or 40828 for intermittent peritoneal dialysis |

| Prior organ transplantation |  |
| --- | --- |
| ICD-10-GM Codes: |  |
| Z94 | Transplanted organ and tissue status |
| OPS Codes: |  |
| 5-504 | Liver transplantation |
| 5-375 | Heart and heart-lung transplantation |
| 5-555 | Kidney transplantation |
| 5-335 | Lung transplantation |
| 5-5281 | Transplantation of a pancreas segment |
| 5-5282 | Transplantation of the pancreas (whole organ) |
| 5-4676 | Small intestine transplantation |

| Prior major surgery |  |
| --- | --- |
| OPS Codes: |  |
| 5-32 | Excision and resection of lung and bronchus |
| 5-33 | Other operations on lung and bronchus |
| 5-34 | Operations on the chest wall, pleura, mediastinum and diaphragm |
| 5-35 | Operations on the valves and septa of the heart and pericardial vessels |
| 5-36 | Operations on the coronary vessels |
| 5-37 | Surgical treatment of arrhythmias and other operations on the heart and pericardium |
| 8-851 | Bypass surgery (using the heart-lung machine) |
| 5-38 | Incision excision and occlusion of blood vessels. |
| 5-39 | Other operations on blood vessels |
| 5-42 | Surgery on the oesophagus |
| 5-43 | Incision, excision and resection of the stomach |
| 5-44 | Extended stomach resection and other operations on the stomach |
| 5-45 | Incision, excision, resection and anastomosis of the small and large intestine |
| 5-46 | Other operations on the small and large intestine |
| 5-47 | Operations on the appendix |
| 5-48 | Operations on the rectum |
| 5-49 | Operations on the anus |
| 5-50 | Operations on the liver |
| 5-51 | Operations on the gallbladder and bile ducts |
| 5-52 | Operations on the pancreas |
| 5-53 | Abdominal hernia repair |
| 5-54 | Other operations in the abdominal region |
| 5-78 | Operations on other bones |
| 5-79 | Reduction of fractures and dislocations |
| 5-80 | Open joint surgery |
| 5-81 | Arthroscopic joint surgery |
| 5-82 | Prosthetic joint and bone replacement |
| 5-83 | Operations on the spine |
| 5-84 | Operations on the hand |
| 5-85 | Operations on muscles, tendons, fasciae and bursae |
| 5-86 | Replantation, exarticulation and amputation of extremities and other operations on the organs of locomotion |
| 5-01 | Incision (trepanation) and excision of the skull, brain and meninges |
| 5-02 | Other operations on the skull, brain and meninges |
| 5-03 | Operations on the spinal cord, spinal meninges and spinal canal |
| 5-04 | Operations on the nerves and nerve ganglia |
| 5-05 | Other operations on the nerves and nerve ganglia |
| 5-55 | Operations on the kidneys |
| 5-56 | Operations on the ureters |
| 5-57 | Operations on the bladder |
| 5-58 | Operations on the urethra |
| 5-59 | Other operations on the urinary organs |
| 5-60 | Operations on the prostate and seminal vesicles |
| 5-61 | Operations on the scrotum and tunica vaginalis testis |
| 5-62 | Operations on the testicles |
| 5-63 | Operations on the spermatic cord, epididymis and vas deferens |
| 5-64 | Operations on the penis |
| 5-65 | Operations on the ovary |
| 5-66 | Operations on the fallopian tubes |
| 5-67 | Operations for facial bone fractures |
| 5-68 | Incision, excision and removal of the uterus |
| 5-69 | Other operations on the uterus and operations on the parametria |
| 5-70 | Operations on the vagina and recto-uterine pouch |
| 5-71 | Operations on the vulva |
| 5-72 | Childbirth with breech presentation and instrumental delivery |
| 5-73 | Other operations to induce labour and during the birth |
| 5-74 | Caesarean section and child development |
| 5-75 | Other obstetric operations |
| 5-87 | Excision and resection of the breast |
| 5-88 | Other operations on the breast |
| 8-989 | Surgical complex treatment in cases of severe infection |

| Prior palliative treatment |  |
| --- | --- |
| ICD-10-GM Codes: |  |
| Z51.5 | Palliative care |
| OPS: |  |
| 8-982 | Palliative medical complex treatment |
| 8-98e | Specialized inpatient palliative medical complex treatment |
| 8-98h | Specialized palliative medical complex treatment through a palliative care service |
| Statutory scale of fees for physicians (GOÃ„) Codes: |  |
| 1425 | Initial care in specialized outpatient palliative care |
| 1426 | Follow-up prescription for continuation of the specialized outpatient palliative care |
| 3370 | Palliative medical initial diagnosis |
| 3371 | Supplementary fee for palliative medical care in the medical practice |
| 3372 | Supplementary fee for palliative medical care in the home |
| 3373 | Supplementary fee for palliative medical care in the home |
| 1425 | Initial prescription for specialized outpatient palliative care |
| 1426 | Follow-up prescription for continuation of the specialized outpatient palliative care |
| 3370 | Palliative medical initial diagnosis of patient status including treatment plan |
| 3371 | Supplementary fee to the insured persons flat rate 03000 for palliative medical care of the patient in the medical practice |
| 3372 | Supplementary fee to Catalogue of Tariffs for Physicians code 01410 or 01413 for palliative medical care in the home |
| 3373 | Supplementary fee to Catalogue of Tariffs for Physicians code 01411, 01412 or 01415 for palliative medical care in the home |
| 4370 | Palliative medical initial diagnosis |
| 4371 | Supplementary fee to the insured persons flat rate 04000 for palliative medical care of the patient in the medical practice |
| 4372 | Supplementary fee to Catalogue of Tariffs for Physicians code 01410 or 01413 for palliative medical care in the home |
| 4373 | Supplementary fee to Catalogue of Tariffs for Physicians code 01411, 01412 or 01415 for palliative medical care in the home |
| 37302 | Supplementary fee to the insured persons flat rate or basic flat rate for the coordinating panel doctor |
| 37314 | Consultation discussion doctor with an additional designation palliative medicine |
| 37318 | Telephone consultation |
| 37300 | Palliative medical initial diagnosis of patient status including treatment plan |
| 37305 | Supplementary fee to tariff codes 01410 and 01413 for palliative medical care in the home |
| 37306 | Supplementary fee to tariff codes 01411 01412 and 01415 for palliative medical care in the home |
| 37317 | Supplementary fee to tariff code 37302 for accessibility and willingness to visit in critical phases |
| 37320 | Case conference |

| Pre-existing asplenia |  |
| --- | --- |
| coded in the five years prior to sepsis index hospitalization |  |
| ICD-10-GM Codes: |  |
| Q89.0 | Asplenia (congenital) |
| Q89.01 | Asplenia (congenital) |
| OPS Codes: |  |
| 5-413.1 | Splenectomy, total |
